# Differences between images of large adenoma and protruding type of gallbladder carcinoma

**DOI:** 10.3892/ol.2013.1248

**Published:** 2013-03-12

**Authors:** WANGXUN JIN, CHENGWU ZHANG, XIAODONG HE, YUYUN XU, LI WANG, ZHONGSHENG ZHAO

**Affiliations:** 1 Departments of Hepatology and Biliary Surgery, Zhejiang Cancer Hospital, Hangzhou 310000, P.R China; 2 Radiology, Zhejiang Cancer Hospital, Hangzhou 310000, P.R China; 3 Sonography, Zhejiang Cancer Hospital, Hangzhou 310000, P.R China; 4 Pathology, Zhejiang Provincial People’s Hospital, Hangzhou 310000, P.R China; 5 Department of Abdominal Tumor Surgery, Zhejiang Cancer Hospital, Hangzhou 310000, P.R China

**Keywords:** polypoid lesion of gallbladder, neoplasm, diagnosis, computed tomography, magnetic resonance

## Abstract

The aim of this study was to investigate the differences between images of large adenoma of the gallbladder and the protruding type carcinoma of the gallbladder. A retrospective study was performed on 130 patients who underwent cholecystectomy or biopsy for gallbladder polypoid lesions larger than 10 mm; among them, 20 patients were malignant and 110 patients were benign. Patients’ details including ultrasonography (US), computed tomography (CT) and magnetic resonance (MR) findings were analyzed. All patients whose lesions were >15 mm by US, had CT or MR scans to further determine the nature of the lesion; two patients who were suspected to have a malignant lesion due to their large tumor size were benign by histological examination. Distinct differences were found between large adenoma and protruding type of gallbladder carcinoma. There were distinct differences between adenomas and the protruding type gallbladder cancers, and there was a pathological basis for the differences. Benign tumors had a more homogeneous texture, had spaces between the tumor and the gallbladder wall and a relatively normal configuration of the gallbladder wall. Based on these findings, certain lesions could be definitively diagnosed as benign adenomas and could help in treatment strategy.

## Introduction

The detection of polypoid lesions of the gallbladder (PLGs) has increased significantly due to the widespread use of ultrasonography (US) imaging techniques (1–3). US is the first choice in the diagnosis of this disease and enhanced computed tomography (CT) and magnetic resonance imaging (MRI) confirm its nature (4–8). Many studies have indicated that the size of tumor is vital for diagnosis. A diameter >10 mm is regarded as the threshold for malignancy (3,9–11). In this study, a large tumor size did not necessarily mean that the lesion was malignant; the configuration of the tumor and the gallbladder wall and the existence of space between the two were the main indicators of the nature of the lesion. This knowledge will enable optimal management of this type of patient. In this article, a retrospective analysis of clinical data of PLG patients over a 5-year time period was performed.

## Materials and methods

### Patients

The study was approved by the Ethics Committee of Zhejiang Provincial People’s Hospital, Hangzhou, China. The study was retrospective so consent was not required. Two qualified radiologists reviewed images from 130 patients from 2005 to 2011 with PLGs larger than 10 mm who consented to the removal of the gallbladder or biopsy. All patients were pathologically diagnosed by routine postoperative examination; the age of patients ranged from 28 to 82 years old.

### CT, MR and US protocol

Patients fasted for at least 8 h before examination; no oral contrast medium or water was administered. CT examinations were performed using Phlilips Brilliance 16 (Philips Medical Systems, Best, Netherlands). Each patient received 100 ml of a nonionic contrast material (iopromide; Ultravist 370, Bayer HealthCare, Berlin, Germany) through an 18-gauge angiographic catheter inserted into a forearm vein. CT scans were routinely obtained with the patient in a supine position. The contrast material was injected at a rate of 3 ml/sec with an automatic power injector. Magnetic resonance cholangiopancreatography was taken using a Siemens 3.0 Tesla MR magnet (Megnetom Trio; Siemens AG, Erlangen, Germany); sonography was performed using Esaote MyLab 70 (Esaote SPA, Genova, Italy) with 1–8 MHz linear array probes or a Toshiba (Applio XG 790; Toshiba, Tokyo, Japan) with 3–6 MHz 6C1 probes. Specific attention was given to the size and the base of the lesion, the space between the lesion and the wall, and the configuration of the wall to elucidate the differences between benign and malignant lesions.

## Results

### Patient characteristics

Twenty-two patients were diagnosed as malignant by CT and 20 of them were confirmed by biopsy or postoperative pathological examination. Two patients with lesions larger than 50 mm were suspected to be malignant preoperatively but were benign on histological examination. One patient had two polypoid lesions. On pathological examination one was benign and the other was malignant (Table I).

### Differences in morphology between large adenoma and protruding type cancer

**Figure 1:**
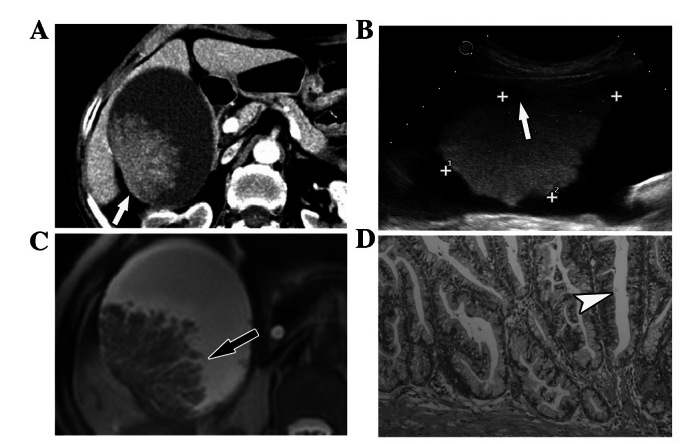
Images from a 69-year-old female with a ten-year history of systemic lupus erythematosus (SLE). (A) The computed tomography (CT) scans show the gallbladder wall running smoothly and naturally (white arrow). (B) Ultrasonography (US) scans show a huge mass, with narrow base and space between the wall and the mass (white arrow). (C) Magnetic resonance (MR) shows the mass resembled a dendritic process with narrow spaces between the processes (black arrow). (D) Villous adenoma; the white arrowhead indicates the narrow space between the villis; H&E staining; magnification, ×100.

**Figure 2:**
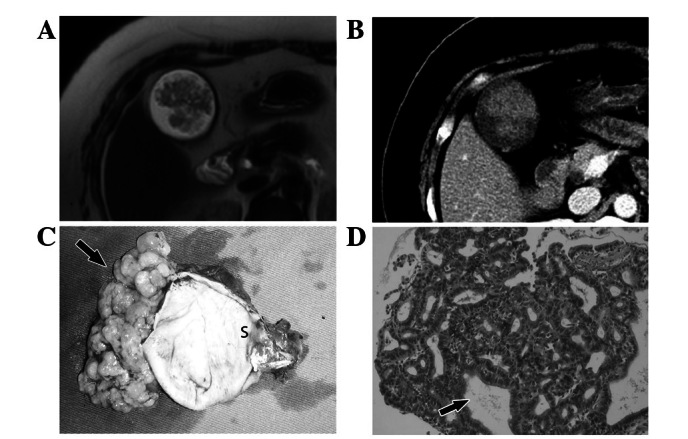
Images from a 50-year-old female. (A) Magnetic resonance (MR) imaging reveals a mass with irregular spaces which appears to float in the gallbladder with a smooth and thin wall. (B) Computed tomography (CT) scans of the same patient. (C) The resected gallbladder (black arrow, mass has an appearance similar to a congregation of mulberries. S, stone obstructs the cyst duct). (D) Tubular adenoma; the black arrow indicates the irregular space between the glands; H&E staining; magnification, ×100.

**Figure 3:**
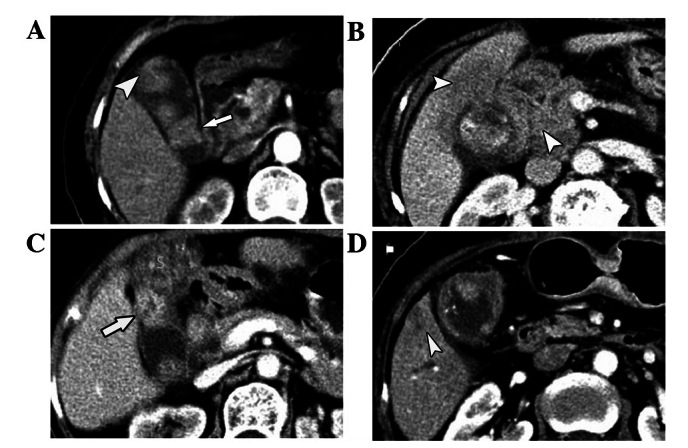
Computed tomography (CT) images of gallbladder cancer. In all cases the tumors were solid, the base of the tumor was wide and the demarcation between the tumor and wall was obscure. (A) Arrowhead and arrow show multiple tumors with wide base; the white arrow shows the tumor-infiltrated and contracted wall. (B) Gallbladder cancer which infiltrated the neighboring tissue (white arrowhead). The pathological diagnosis was obtained by biopsy. (C) White arrow indicates the infiltrated wall and the disappearance of the space between the wall and liver. (D) Arrowhead indicates the metastasis in the liver parenchyma.

**Figure 4:**
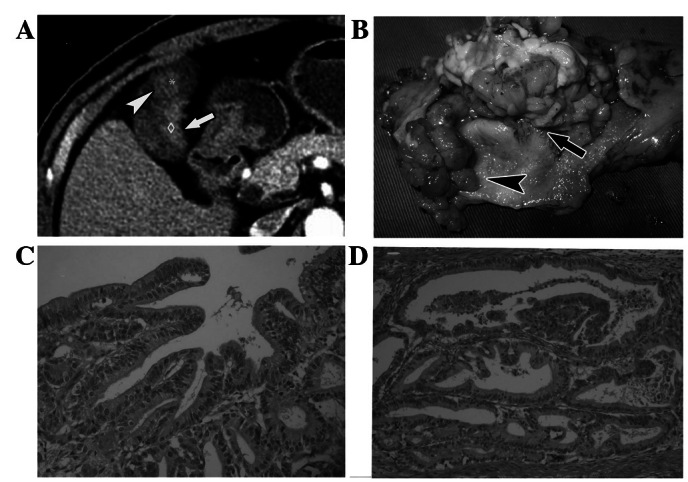
Images from a 67-year-old male whose blood CA19-9 was 139.7 U/ml. (A) Computed tomography (CT) shows there were two tumors in the gallbladder, one had less contact with the gallbladder wall (white arrowhead); another had a wide base with a thickened wall (white arrow; Housefield unit value: ^*^, 50; ^⋄^, 80). (B) Resected gallbladder, benign tumor (black arrowhead) and malignant tumor with thickened and rigid wall (black arrow). (C) Histological result of the benign tumor, tubular adenoma and mild dysplasia (H&E, ×100). (D) Pathological examination of the malignant tumor: moderate differentiated adenocarcinoma (H&E staining; magnification, ×100).

Before the operation, 2 patients were diagnosed with gallbladder cancer due to the large size of their tumors but these were revealed to be adenoma. One was tubular adenoma and the other was rare villious adenoma. Following CT scans, the villious adenoma turned out to be akin to dendritic processes; there were spaces within the mass, the surface had no contraction and the whole shape appeared to be natural like dendritic processes in the MRI scan (Fig. 1). The CT and MRI image of the tubular adenoma was different from that of villious adenoma, the appearance was similar to a congregation of mulberries (Fig. 2). In contrast, the surface of the malignant tumor was uneven, the enhancement of the solid entity of the tumor seemed to be inhomogenous and grew in a solid mass (Fig. 3). In Fig. 4, where a benign and malignant tumor grew together in the same gallbladder, the malignant tumor became more enhanced than the benign one (Fig. 4).

### Difference in the base size of the tumor, and the space between the tumor and the gallbladder wall

**Figure 5:**
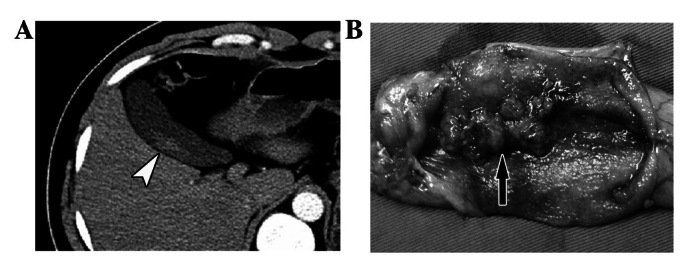
Tubular adenoma from a 28-year-old male. (A) Computed tomography (CT) image shows a lesion parallel with the wall of the gallbladder. There is space between the mass and wall and clear space between the gallbladder and the liver (white arrowhead). (B) Gallbladder removed by laparoscopy (black arrow, narrow polyp with a tiny base).

**Figure 6:**
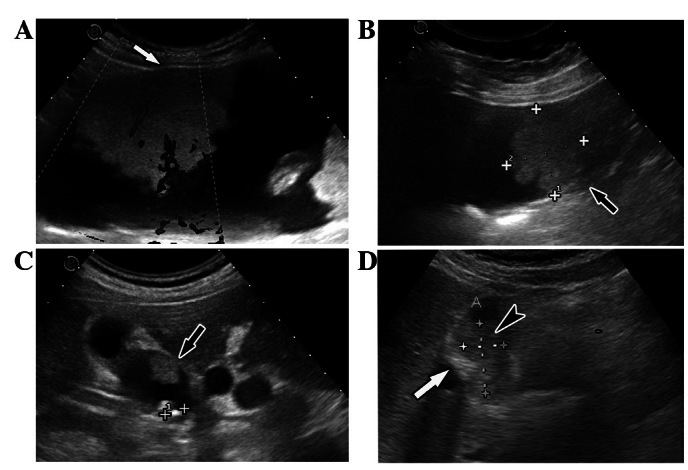
Ultrasonography (US) of adenomas of the gallbladder. The arrows show the wall of the gallbladders were thin and ran smoothly, and the demarcation between the wall and mass was clear. The black arrow in image (C) shows the mass had a narrow base and there was space between the mass and wall. (D) US of gallbladder carcinoma. The white arrow indicates the thickened wall and dotted lines indicate the size of the mass. The base was wide and the demarcation between the wall was obscure.

The base of the benign polypus was smaller than the malignant one and there was space between the tumor and the gallbladder wall. In the case of tubular adenoma, the CT and MR images showed that the mass had almost no contact with the gallbladder wall and the whole mass appeared to be floating in the water. Macroscopically, the mass was connected to the wall of the gallbladder with small and slender fibers and could easily be removed from the wall of the gallbladder, while the gallbladder wall remained smooth with integrity (Fig. 2). In the case where benign and malignant tumors coexisted, there was a space between the wall and the tumor, while the space disappeared between the malignant tumors and the wall (Fig. 4). In the case where the lesion was a polypoid with a slender pedicel, the CT scan showed a strip parallel with the wall resembling a trailing plant, and was separated from the wall by a narrow space (Fig. 5). In contrast, the base of the protruding carcinoma was wide, the space between the polypus and the gallbladder wall disappeared and the enhancement became more evident close to the base of the tumor (Figs. 4 and 6).

### Shape of the gallbladder wall

The gallbladder wall with the benign tumor was smooth and soft, had the normal thickness and normal extent of enhancement, with the normal space between the wall and the liver. The wall of malignant lesions, however, became thicker and stiff and the space became obscure. In some cases it disappeared. In certain cases, the wall had broken and the lesion had infiltrated the liver parenchyma, which was a sign of a malignant lesion.

## Discussion

Polypoid lesions of the gallbladder are an imaging feature, which indicate a wide variety of pathology. They affect ∼5% of the adult population (3,12,13). Although most of these lesions are benign, some early carcinomas of the gallbladder present as polypoid lesions; malignant transformation is always a concern. The differentiation of benign and malignant lesions can be challenging. Several features, including patient age, tumor size and the speed of growth of the lesions, are important discriminating features between benign and malignant polyps. Many studies have shown that age >50 years and a polyp size >1 cm are the two most important factors predicting malignancy in polypoid lesions of the gallbladder (3,9–11).

Microscopically, malignant tumors no longer possess the normal structure of the original tissue. There is frequent necrosis and subsequent repair within the tumor; the surface of a malignant tumor is often uneven and the whole configuration is unnatural and becomes stiff (14). Growing in an infiltrative way, the malignant tumors invade neighboring tissue; thus, the demarcation between the tumor and the normal tissue is obscure. Benign tumors do not grow uncontrollably; most benign tumors have a similar structure to the tissue they originate from and do not invade neighboring tissues and spread throughout the body. Since adenoma has the structure of gland tube, which is characterized by short fibrovascular stalks that are covered by dysplastic or neoplastic cells, the CT and MR images of large adenoma have the similar shapes, akin to float grass. In contrast, gallbladder cancer infiltrates the base and there is necrosis on the inside of the tumor and the image appears to be inhomogenous without a natural texture. Macroscopically, the gallbladder tumors are classified into the protruding, flat or infiltrative type (15). US, CT and MR images are used to classify these types (8,16–20).

In this study, pathological differences in the shape and texture of the mass, the base of the mass, the space between the tumor and gallbladder wall, and the changes of the gallbladder wall were used to determine whether the lesion was benign or malignant. The results support the differences between large adenoma and protruding type carcinoma. In gallbladder adenoma, even though the lesions were very large there were still spaces within the tumor, indicating that the tumor was not a solid mass; in the villous adenoma, the spaces were narrow and resembled dendritic processes; in the tubular adenoma, the spaces were irregular. Microscopically, this kind of spaces exist between the fibrovascular stalks that are covered with gland. Since the malignant tumor grows in an infiltrative way and always has a wide base, there is no space between the tumor and the gallbladder wall and the gallbladder walls are always thickened and contracted; the spaces between the gall-bladder wall and the liver disappear when the tumors break the gallbladder wall and invade the liver parenchyma.

Many studies have demonstrated that lesion size and patient age are strong indicators of a malignant tumor. In this study, the large size of polypoid lesions of the gallbladder did not necessarily mean the tumor was malignant. A possible reason for this may be that the cancerous change needs time and the speed of this change is relatively slow. Conversely, small adenoma does not always mean benign. According to a study conducted by Roa *et al*(21), adenomas associated with cancer may measure <5 mm. Using polyp size to decide surgical behavior may, therefore, be misleading. Adenoma is a precancerous disease which could transform to adenocarcinoma in an adenoma-dysplasia-cancer manner (22,23). A protruding type of gallbladder was likely the result of adenoma under continuing stimuli such as gallstones or chronic inflammation. In this study, the co-existence of a benign and malignant tumor in one patient was evidence of this theory.

Although it is difficult to discriminate between large adenoma and cancer, removing the gallbladder by open laparotomy is usually the first choice of treatment for these two diseases. However, in an era of minimally invasive surgery, both doctors and patients prefer to choose laparoscopic cholecystectomy. Size may not be the key indicator to discriminate benign from malignant lesions, or the indication for a laparoscopic or open procedure. Preoperative images indicate that the feasibility of laparoscopic cholecystectomy mainly depends on the lesion’s configuration; they should be confined to the lumen of the gallbladder, the wall of the gallbladder should be smooth and thin and the space between the wall and liver should be clear; this indicates that the separation of the gallbladder from the liver should be relatively simple. Many articles have reported that even PLGs with a diameter >1.5 mm, a potential early-stage cancer, could still be resected by laparoscopic cholecystectomy with full-thickness dissection (24–26). The key issue with such surgery is to maintain the integrity of the gallbladder and protect the Trocar port in order to avoid the dissemination of the cancer in case the lesion is malignant (27,28).

## Figures and Tables

**Table I:** Patients’ clinical data.

Characteristics	No.	Percentage
Total (n=130)		
Benign	110/130	85
Malignant	20/130	15
Malignant (n=20)		
Platform	13/20	65
Protruding lesions	7/20	35
Female	16/20	80
Male	4/20	20
Benign (n=110)		
Lesion >20 mm	8/110	7
Lesion >50 mm	2/110	1.8
Laparoscopic cholecystectomy	102/110	93
Open cholecystectomy	8/110	7
